# A simple prediction model to estimate obstructive coronary artery disease

**DOI:** 10.1186/s12872-018-0745-0

**Published:** 2018-01-16

**Authors:** Shiqun Chen, Yong Liu, Sheikh Mohammed Shariful Islam, Hua Yao, Yingling Zhou, Ji-yan Chen, Qiang Li

**Affiliations:** 1 Department of Cardiology, Provincial Key Laboratory of Coronary Heart Disease, Guangdong Cardiovascular Institute, Guangdong General Hospital, Guangdong Academy of Medical Sciences, Guangzhou, 510100 China; 2 Guangdong General Hospital Zhuhai Hospital, Guangdong Academy of Medical Sciences, Zhuhai, 519000 China; 3 The George Institute for Global Health, University of Sydney, Camperdown, NSW 2050 Australia

**Keywords:** Prediction model, Obstructive coronary artery disease, Framingham risk

## Abstract

**Background:**

A simple noninvasive model to predict obstructive coronary artery disease (OCAD) may promote risk stratification and reduce the burden of coronary artery disease (CAD). This study aimed to develop pre-procedural, noninvasive prediction models that better estimate the probability of OCAD among patients with suspected CAD undergoing elective coronary angiography (CAG).

**Methods:**

We included 1262 patients, who had reliable Framingham risk variable data, in a cohort without known CAD from a prospective registry of patients referred for elective CAG. We investigated pre-procedural OCAD (≥50% stenosis in at least one major coronary vessel based on CAG) predictors.

**Results:**

A total of 945 (74.9%) participants had OCAD. The final modified Framingham scoring (MFS) model consisted of anemia, high-sensitivity C-reactive protein, left ventricular ejection fraction, and five Framingham factors (age, sex, total and high-density lipoprotein cholesterol, and hypertension). Bootstrap method (1000 times) revealed that the model demonstrated a good discriminative power (c statistic, 0.729 ± 0.0225; 95% CI, 0.69–0.77). MFS provided adequate goodness of fit (*P* = 0.43) and showed better performance than Framingham score (c statistic, 0.703 vs. 0.521; *P* < 0.001) in predicting OCAD, thereby identifying patients with high risks for OCAD (risk score ≥ 27) with ≥70% predictive value in 68.8% of subjects (range, 37.2–87.3% for low [≤17] and very high [≥41] risk scores).

**Conclusion:**

Our data suggested that the simple MFS risk stratification tool, which is available in most primary-level clinics, showed good performance in estimating the probability of OCAD in relatively stable patients with suspected CAD; nevertheless, further validation is needed.

**Electronic supplementary material:**

The online version of this article (10.1186/s12872-018-0745-0) contains supplementary material, which is available to authorized users.

## Introduction

A report from the national cardiovascular data registry of the American College of Cardiology showed that only 41% of patients undergoing elective coronary angiography (CAG) are diagnosed as having obstructive coronary artery disease (OCAD); hence, a better risk stratification to increase pretest probability of coronary artery disease (CAD) appears warranted [1]. A recent meta-analysis (33 studies, 120,548 participants) suggested that OCAD in patients with acute coronary syndrome has a significantly higher cardiovascular risk at baseline and a higher likelihood of death or major cardiovascular events [2].

The classic risk stratification tool for CAD was the Framingham score system, which predicts the 10-year risk of coronary heart disease; however, the association of Framingham score with plaque burden is less robust [3, 4]. Although non-invasive diagnostic technology advancements, such as stress testing and computed tomography (CT) scanning adopted to increase the pretest probability of CAD in most tertiary hospitals, are available, high costs and unavailability limit their application in daily clinical practice. Ibrahim et al. recently established a new clinical and biomarker score with high accuracy for predicting the presence of anatomically significant CAD (≥70% stenosis), which included clinical variables (male sex and previous percutaneous coronary intervention (PCI)) and four biomarkers (midkine, adiponectin, apolipoprotein C-I, and kidney injury molecule-1), among patients with known CAD (e.g., patients with previous acute myocardial infarctions (MI), who had PCI, or who underwent coronary artery bypass grafting (CABG)). However, whether this model could predict CAD in patients presenting at primary-level hospitals or clinics is unknown [5].

Therefore, this study aimed to establish a new simple prediction model, including traditional Framingham risk factors for OCAD, based on a continuously recruited at-risk cohort from an observational database investigating acute kidney injuries following elective CAG [6]. We hypothesized that the addition of new contemporary predictors to traditional Framingham risk factors could increase the accuracy of predicting anatomically significant CAD and, consequently, the novel model could be used in a broader population with suspected CAD.

## Methods

### Study population

The study population was derived from a prospective observational study PRECOMIN (NCT01400295) database between January 2010 and December 2013. The PRECOMIN study aimed at identifying the optimal contrast volume to prevent contrast-induced nephropathy among 3237 consecutive patients undergoing PCI/CAG, [6]. Patients who had previous PCI or previous MI, CABG, acute MI, or emergent PCI and those who lack total cholesterol (CHO) or high-density lipoprotein cholesterol (HDL-C) data were excluded (Additional file 1: Figure S1).

A total of 1262 patients without known CAD undergoing elective CAG were included in the analysis. The Institutional Ethics Research Committee approved the study, and all patients provided written informed consent.

### Coronary angiography and data collection

CAG or PCI was performed according to the interventional cardiologist’s preference, which is guided by institutional policy and practice. Baseline data, angiographic characteristics, and medication data were prospectively defined and have been reported in a previous study [6]. During CAG, the highest percent stenosis value within each major coronary artery or their branches was recorded. High-sensitivity C reactive protein was tested with a Beckman Coulter Immage immunobiochemistry system (USA) using nephelometry (unit: mg/L). Left ventricular ejection fraction was calculated by using the biplane modified Simpson’s rule by Two-Dimensional Echocardiography.

### Definition

The primary endpoint of our study was OCAD, which was defined as (≥50% stenosis by diameter in at least one major coronary vessel based on CAG) [2]. Anemia was defined according to the World Health Organization criteria, i.e., baseline hematocrit value < 39% for men and < 36% for women [7]. Hypertension was definite present if the participant was under treatment with antihypertensive medication or systolic≥140 mmHg or diastolic ≥90 mmHg [3].

### Statistical analysis

Continuous variables were compared with the t-test, and categorical variables were compared with the chi-square test or Fisher exact test, as appropriate. A logistic regression model was developed to predict OCAD in the PRECOMIN substudy. Significant predictors from the univariate analysis and non-significant variables with potential clinical relevance, including traditional Framingham risk factors, were evaluated as candidate factors (age, sex, smoking status, diabetes, CHO, high-density lipoprotein cholesterol [HDL-C], hypertension, baseline systolic blood pressure, and diastolic blood pressure, high-sensitivity C reactive protein [hs-CRP],left ventricular ejection fraction [LVEF], anemia, weight, serum creatinine, serum albumin, uric acid,HbA1c, Lp(a), blood urea nitrogen,) to determine their association with OCAD in a multivariable model [3]. Collinearity and interaction between variables were also evaluated. Backward elimination approach was employed to create a reduced model by successively removing non-significant covariates until all the remaining predictors (age, sex, CHO and HDL-C, hypertension, smoke, anemia, hs-CRP and LVEF) are statistically significant (*P* < 0.1). Then, we manually investigated the contribution of the remaining predictors to find out the final predictors including age, sex, CHO and HDL-C, hypertension, anemia, hs-CRP, LVEF. We used regression coefficients from the model to generate point scores for predicting the probability of OCAD [8, 9]. The final prediction model was assessed using the area under the receiver operating characteristic (ROC) curve and concordance c-statistic for discriminative ability, and the Hosmer–Lemeshow goodness-of-fit statistic for calibration using fifths of the fitted risk values [10]. Moreover, the final model was tested by bootstrapping method (1000 times) to evaluate the stability of the c-statistics. We assumed that missing data occurred randomly, depending on the clinical variables and the CAG results. We performed multiple imputations using Markov chain Monte Carlo (MCMC) and fully conditional specification (FCS) [11–13]. Comparisons of baseline characteristics and OCAD incidence between patients with and those without missing values were performed [14, 15]. A *P* value < 0.05 was considered statistically significant in all tests. All analyses were performed using SAS version 9.3 (SAS Institute, Cary, NC).

## Results

A total of 1262 patients with complete data were included in the final analysis (Additional file 1: Figure S1). The study subjects’ baseline characteristics, dichotomized as a function of the presence or absence of significant CAD, are detailed in Table 1. Numerous baseline characteristics differed between patients with (945, 74.9%) and those without OCAD.

**Table 1:** Baseline characteristics of patients without and those with obstructive coronary artery disease

Variables	No (%) of patients with available data	Subjects without coronary stenosis ≥50%(*n* = 317)	Subjects with coronary stenosis ≥50% (*n* = 945)	*P* value
Demographic				
Age	1262(100)	62.0 ± 10.4	64.2 ± 10.2	< 0.001
Gender, men	1262(100)	181(57.1%)	693(73.3%)	< 0.001
Signs and measurement				
Heart rate, beats/min	1261(99.9)	73.4 ± 11.3	72.9 ± 11.9	0.49
Systolic BP, mm Hg	1262(100)	131.9 ± 17.9	132.7 ± 18.6	0.54
Diastolic BP, mm Hg	1262(100)	78.3 ± 12	77.5 ± 11.6	0.32
Weight, kg	1257(99.6)	63.5 ± 9.5	65.2 ± 10.9	0.01
Medical history				
Smoking	1262 (100)	80(26.2%)	347 (36.7%)	< 0.001
Hypertension	1262(100)	152(48.0%)	615 (65.1%)	< 0.001
Congestive heart failure	1262(100)	19(6.5%)	79 (8.4%)	0.29
Diabetes mellitus	1260(99.8)	51(16.1%)	259 (27.5%)	< 0.001
Hyperlipidemia	1262(100)	42(13.3%)	144 (15.2%)	0.75
Anemia	1262(100)	79(25.2%)	349 (37.5%)	< 0.001
Medications				
ACEI/ARB	1262(100)	240(75.7%)	864(89.5%)	< 0.001
Diuretics	1261(99.9)	36(11.4%)	91(9.6%)	0.38
β-blocker	1262(100)	244(76.9%)	853(90.0%)	< 0.001
Statin	1262(100)	279 (88.0%)	912(96.5%)	< 0.001
Calcium-channel blocker	1259(99.8)	60(19.0%)	198(21.0%)	0.44
Physical examination				
LVEF, %	1013(80.3)	64.6 ± 11	61.4 ± 11.9	< 0.001
Laboratory measures				
Total cholesterol, mg/dl	1262(100)	168.2 ± 38.8	172.6 ± 64.6	0.15
HDL-C,mg/dl	1262(100)	40 ± 10.8	36.8 ± 10.3	< 0.001
Triglyceride, μmol/l	1262(100)	2.3 ± 11.8	1.6 ± 1.4	0.27
LDL cholesterol, μmol/l	1262(100)	2.5 ± 0.8	2.6 ± 0.9	0.19
Lp (a), μmol/l	1081(85.7)	211.4 ± 231.1	287.2 ± 311.4	< 0.001
Blood urea nitrogen, mg/dl	1246(98.7)	4.7 ± 1.6	5.2 ± 2.3	< 0.001
Serum Creatinine, μmol/l	1261(99.9)	78.0 ± 29.6	89.7 ± 50.7	< 0.001
Hemoglobin, g/ml	1246(100)	135.1 ± 15.1	133.3 ± 16.0	0.0929
Serum albumin, g/l	1220(96.7)	36.9 ± 4.1	36.1 ± 3.9	0.0035
Urine PH	1213(96.1)	5.9 ± 0.7	6.0 ± 0.7	0.48
HbA1c, %	1043(82.6)	6.3 ± 1.1	6.5 ± 1.2	0.006
Hs-CRP, mmol/l	849(67.3)	3.8 ± 5.7	6.6 ± 13.0	< 0.001
Uric acid, mmol/l	994(78.8)	364.6 ± 92.7	389.3 ± 102.5	< 0.001
B-type natriuretic peptide, pg/m	1041(82.5)	609.4 ± 2061.0	772.4 ± 2289.0	0.29

*LVEF* = left ventricular ejection fraction; *ACEI/ARB* = angiotensin-converting enzyme inhibitor/angiotensin receptor blocker; *HDL-C* = high-density lipoprotein cholesterol; *LDL-C* = low-density lipoprotein-cholesterol; *HbA1c* = glycated hemoglobin; *Hs-CRP* = high-sensitivity C-reactive protein

### Final modified Framingham prediction model

The final modified Framingham prediction model consisted of the following: anemia (odds ratio [OR], 1.556; *P* = 0.0053), hs-CRP (OR, 1.029; *P* = 0.0006), LVEF (OR, 0.979; *P* = 0.075), and five Framingham factors (age, sex, CHO and HDL-C, hypertension), and our model equation was “F(y) = − 0.0468 + 0.0204(age) + 1.0961(gender) + 0.5444(hypertension) + 0.0055(CHO)-0.0257(HDL-C)-0.022(LVEF) + 0.5677(anemia) + 0.0254(hs-CRP)”(Table 2).

**Table 2:** Univariate analyses and multivariate associations between variables and obstructive coronary artery disease

Risk factors	Univariate logistic regression^a^			Multivariate logistic regression^b^		
	OR	95% CI	P value	OR	95% CI	P value
Age, (per year)	1.11	1.04–1.18	< 0.01	1.02	1.00–1.04	0.05
Gender (male vs. female)	2.07	1.56–2.69	< 0.01	2.99	1.97–4.45	< 0.01
Hypertension (yes vs. no)	2.02	1.56–2.62	< 0.01	1.72	1.17–2.55	< 0.01
Anemia (yes vs. no)	1.78	1.33–2.37	< 0.01	1.76	1.14–2.72	< 0.01
LVEF (per %)	0.97	0.96–0.99	< 0.01	0.98	0.96–1.00	< 0.01
Hs-CRP (per mmol/l)	1.04	1.01–1.07	< 0.01	1.03	1.00–1.05	0.07
TC (per 10 mg/dl)	1.02	0.99–1.05	0.25	1.06	1.01–1.11	0.03
HDL-C (per mg/dl)	0.97	0.96–0.99	< 0.01	0.98	0.96–0.99	< 0.01

OR: odds ratio; CI: confidence interval; LVEF = left ventricular ejection fraction; Hs-CRP = high-sensitivity C-reactive protein; TC: total cholesterol; HDL-C = high-density lipoprotein cholesterol

^a^Univariate logistic regression analysis was performed in 1262 patients

^b^Multivariate logistic regression analysis was performed in 683 patients without missing data of the variables in the final model

### Modified Framingham scoring (MFS) system

In the entire cohort, the independent OCAD predictors were anemia, hs-CRP, and LVEF, while the Framingham risk factors were age, sex, CHO and HDL-C, and hypertension. Model fitting performed on the validation cohort showed that the MFS model performed better than the traditional Framingham model (Table 3).

**Table 3:** Modified Framingham risk factor and Framingham risk factor model fitting

Model	AUC	AIC	HLG	P value
Modified Framingham risk factors	0.719	691	0.38	Reference
Framingham risk factors	0.693	705	0.35	0.059
Modified Framingham score	0.703	689	0.43	Reference
Framingham score	0.521	753	0.03	< 0.001

AUC: area under the curve; AIC: Akaike information criterion; HLG: Hosmer and Lemeshow goodness-of-fit test

**Fig. 1:**
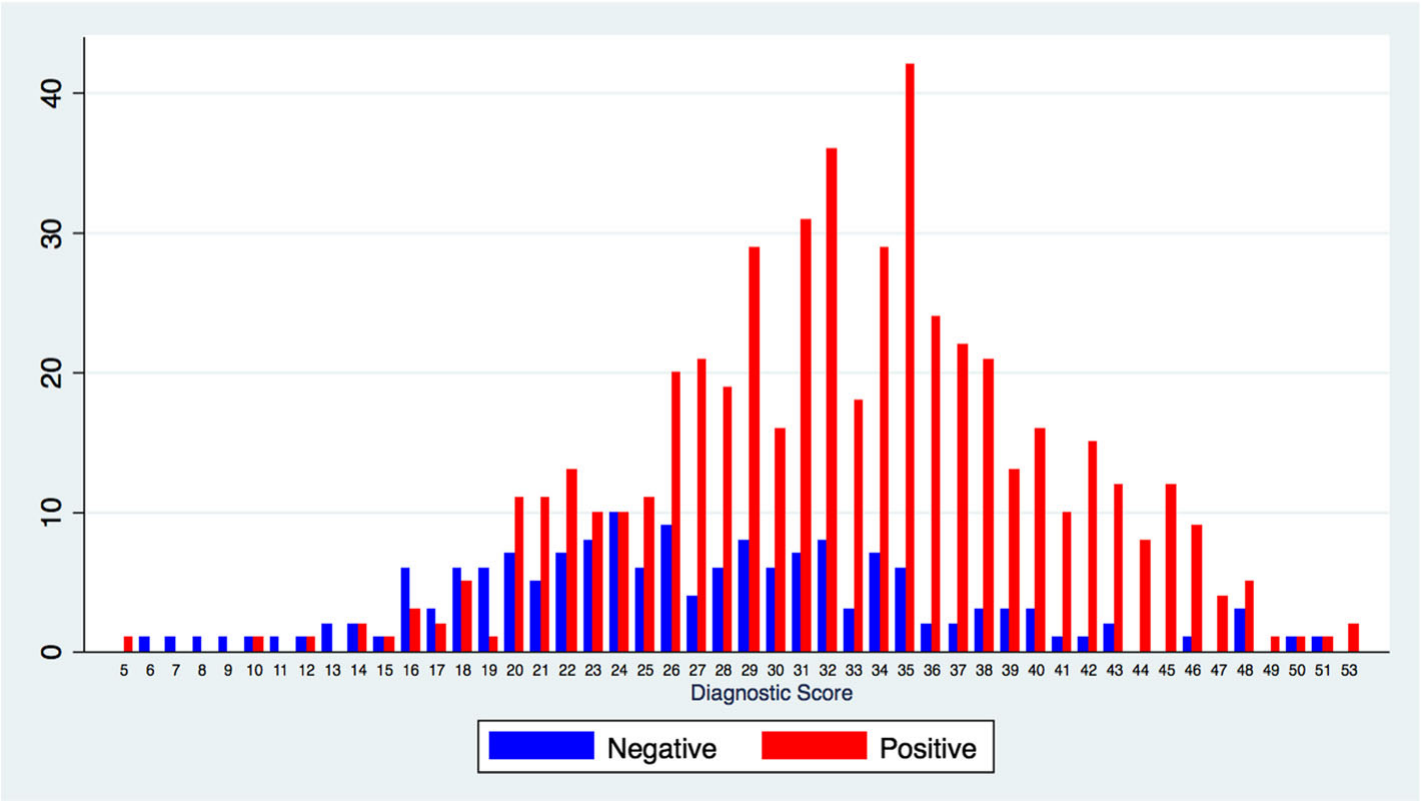
Bimodal Modified Framingham scoring (MFS) distribution

Additionally, individual scores were subsequently calculated for patients in the validation cohort, and the results were expressed as a function of CAD presence. Consequently, a bimodal score distribution was found (Fig. 1), with higher OCAD prevalence in patients with higher scores and lower OCAD prevalence in those with lower scores. Moreover, a bimodal distribution of OCAD score was noted in the validation set (*n* = 683), with preponderance of patients with significant CAD with higher scores (positive: subjects with at least 1 coronary stenosis ≥50%; negative: subjects without coronary stenosis ≥50%).

### Validation by bootstrap method and comparison of risk score

Bootstrap method (1000 times) revealed that the model demonstrated a good discriminative power (c-statistic, 0.729 ± 0.0225; 95% CI, 0.69–0.77).

**Fig. 2:**
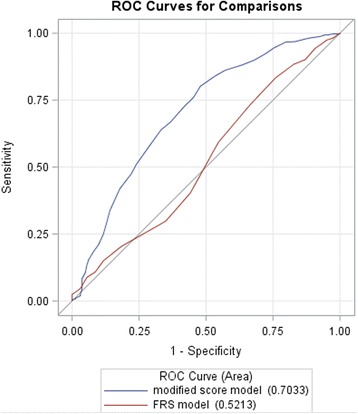
Receiver operating characteristic curve

In addition, the MFS provided adequate goodness of fit (*P* = 0.43). For the operating characteristics of the OCAD algorithm across various scores, the MFS model had a higher area under the ROC curve than the Framingham score (c-statistic, 0.703 vs. 0.521; *P* < 0.001); similarly, the modified Framingham risk factors model had a higher area under the ROC curve than the Framingham risk factors model (c-statistic, 0.719 vs. 0.693; *P* = 0.059) (Table 3; Fig. 2).

**Fig. 3:**
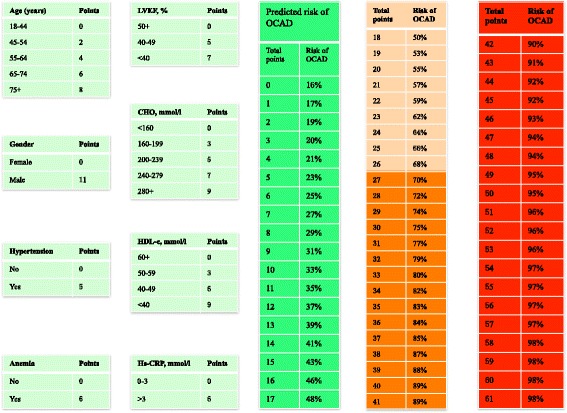
OCAD prevalence according to modified Framingham score

**Fig. 4:**
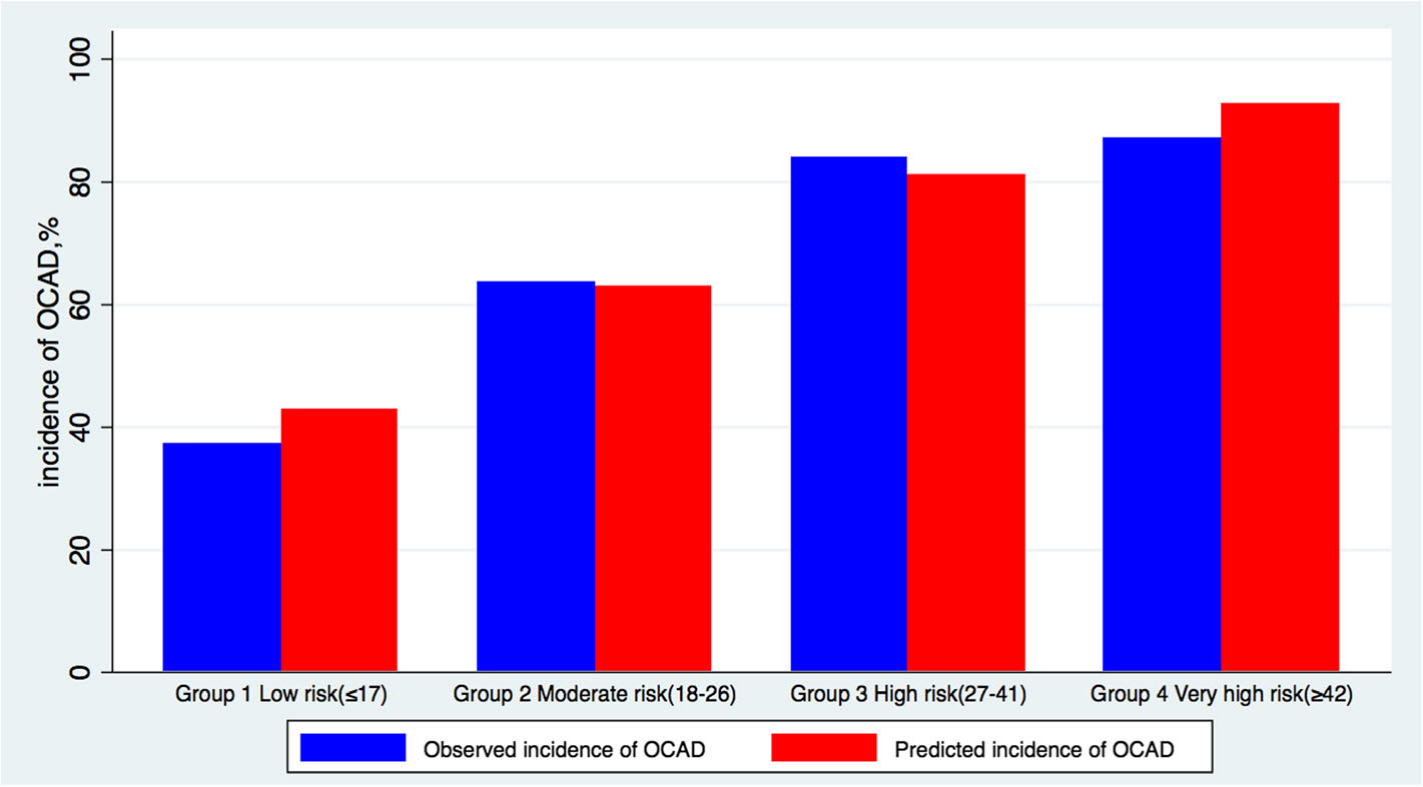
Scheme to define OCAD risk score

Moreover, the score was divided into four categories according to predicted risk (low risk score, ≤17; moderate risk score, 18–26; high risk score, 27–41; and very high risk score, ≥42), which allowed identifying patients with high risk for OCAD (risk score ≥ 27) with ≥70% predictive value in 68.8% of subjects (range, 37.2–87.3% for a low [≤17] and very high [≥41] risk score) (Fig. 3). The predicted risk of CIN in the validation set is presented in parallel to the observed OCAD prevalence in each risk group (Fig. 4).

### Comparison between patients with missing data and those with complete data

Most of the baseline characteristics were similar between patients with missing data (*n* = 579) and those with complete data (*n* = 683). The missing data in the final model included gender, hypertension, smoking, CHO and HDL-C, and LVEF. Patients with missing data were younger and had lower hs-CRP level, systolic BP, and rate of anemia than patients with complete data. No significant difference in OCAD incidence (73.40 vs. 76.13%; *P* = 0.2648) was noted (Additional file 2: Table S1).

### Comparison of patients with complete, MCMC, and FCS imputation data

We performed imputations of clinical variables and CAG results for 100 times using MCMC and FCS. Multivariate logistic regression and ROC curve were analyzed for the final modified Framingham model using complete and imputation data, and we found similar results, including ORs for predictor models and c-statistic by ROC analysis (0.719, 0.712, 0.711) (Additional file 3: Table S2 and Additional file 4: Table S3).

## Discussion

In this study, Framingham risk score showed lower discrimination for OCAD prevalence in patients without known CAD undergoing elective CAG, whereas our new simple MFS model showed better performance in estimating the pretest probability of OCAD and identified more than two thirds of patients at high risk for OCAD.

Framingham risk score including traditional risk factors, which is the classic CAD risk-prediction model, showed less association with OCAD in the present study, which is consistent with a previous Canadian study that investigated the association of Framingham risk score with computed tomography angiography (CTA) measures of coronary atherosclerosis. Coronary atherosclerosis was present in 63.5% of the patients, which suggested a high prevalence similar to that in our study (74.9% of OCAD). Nevertheless, OCAD was diagnosed using CAG in our study, which may have more accuracy and significance for clinical decision than CTA [4].

Our simple modified Framingham risk score (FRS) might outperform FRS by ROC analysis, while we could not further calculate the net reclassification improvement to show how much improvement by using modified FRS to predict OCAD. Because FRS was to predict the 10-year risk of CAD, whose endpoint rate was lower than 15%, however, the modified FRS was to predict the OCAD with higher rate (more than 50%) for patients with suspected CAD, we could not find out ideal overlapping parts, ever after adopting all the cut-off value of endpoint rate [3].

This study established a novel, simple risk stratification method, which could be a useful tool in most primary-level hospitals or clinics and requires no large equipment or expensive examination in identifying patients with high risk for OCAD. Genders et al. established prediction models with a more accurate estimation of the pretest probability of OCAD in lower prevalence populations than that of the Duke clinical score, which is recommended by an American College of Cardiology guideline. However, to improve the model, coronary calcium score by CT scanning and classification of chest pain symptoms (i.e., typical, atypical, or non-specific) are necessary, which requires medical knowledge and experience or equipment and entails costs [16, 17].

Moreover, our results indicated that the use of MFS model suggested the yield of testing (i.e., the proportion of individuals referred for testing who have abnormal results) among patients with a high pretest probability [18]. More expensive or limited diagnostic tests in the risk stratification in a large community population can be addressed by the MFS model. In other word, the simple MFS model will improve the precision of risk stratification for more physicians, to increase invasive procedure among high risk patients, but to reduce invasive procedure among low risk patients.

For OCAD diagnosis, the modified Framingham score had a final area under the ROC curve of 0.703 in the validation set. Although the area under the ROC curves was less than that of previous studies, the performance of the MFS model was good [5, 18]. The small area under the ROC curve in our study could be attributed to the following: first, our study lacked more novel biomarkers, which were selected from nearly one hundred candidate variables [5]; second, the sample size was smaller than that of previous studies [16, 18]; third, we excluded subgroups with essential variables (e.g., PCI) or missed some significant variables (e.g., types of chest pain) [16].

One recent novel prediction score model in America for significant CAD showed better performance (c-statistic, 0.87 in the validation set); at the optimal cut-point, the score was both highly sensitive (77%) and specific (84%) for CAD diagnosis. The model included four biomarkers (midkine, adiponectin, apolipoprotein C-I, and kidney injury molecule-1). However, including these biomarkers in the routine examination in primary-level hospitals or clinics may not be suitable. In addition, the model was established in patients with or without known coronary disease and included previous PCI as a predictor. Our simple model includes traditional risk factors or routine examination without high cost. The development setting was also different, i.e., our simple model was based mostly on Chinese individuals; in the American model, Whites.

The modified Framingham score has three additional variables (i.e., hs-CRP, LVEF, and anemia), which could be associated with both anatomically OCAD and coronary events. A previous meta-analysis including 160,309 people without a history of vascular disease suggested that CRP concentration has continuous associations with the risk of CAD, ischemic stroke, vascular mortality, and death from several cancers and lung diseases that are each of broadly similar size [19]. A Japanese observational study with a median follow-up period of 6.5 years showed that hs-CRP was associated with higher incidence of major adverse cardiac events or all-cause mortality in patients with established CAD and undergoing PCI [20]. The Atherosclerosis Risk in Communities (ARIC) study, which included 14,410 subjects (between 45 and 64 years) without CVD and had a follow-up duration of 6.1 years, showed that anemia is an independent risk factor for CVD outcomes [21]. Another recent cohort study of outpatients with stable CAD (21,829 with baseline hemoglobin levels) showed that anemia is a powerful predictor of cardiovascular and non-cardiovascular mortality [22]. Our study may be the first to identify anemia as a CAD predictor. A prospective study including 100 diagnostic coronary catheterization candidates found that the overall accuracy of akinesia/hypokinesia and LVEF < 55% in predicting abnormal CAG (≥50% stenosis) was poor [23]. Another study on 182 patients undergoing exercise Tl-201 gated single-photon emission computed tomography suggested that worsening of the LVEF during exercise has the potential to detect multivessel CAD among patients without multivessel patterns of reversible defects [24]. In our study, the LVEF measured by average echo could also be a CAD predictor. The predictive value of two new simple predictors (LVEF and anemia) needs further external validation in larger studies. Similarly, the new simple score model also requires further evaluation in relation to risk stratification. In addition, we would investigate the risk factors of dangerous culprit lesions with culprit plaque rupture (CPR) and thin-cap fibro-atheroma (TCFA), such as hypertension, advanced ages, diabetes mellitus or hyperlipidemia, which were evaluated by optical coherence tomography (OCT) or intravenous ultrasound (IVUS) in the futures [25].

### Limitations

Our study has several limitations. First, our study adds significant information to the current literature on pretest probability of OCAD for stable patients without known CAD; however, this is a single-center prospective observational study with a limited sample size. Second, angina types (e.g., classification of chest pain symptoms: typical, atypical, or non-specific) were not considered; thus, the accuracy of the OCAD risk model may have been affected. Thirdly, some bias in patient selection especially in CAG possibly existed; cardiologists tended to recruit patients with more baseline risk factors but with a stable condition for elective CAG. Thus, our findings may not be applicable to patients with low risk for OCAD or with emergent conditions. Fourthly, our study only focused on the anatomical result of angiography without kind of plaque evaluated with imaging (OCTs or IVUS), such as culprit plaque rupture (CPR) and thin-cap fibro-atheroma (TCFA), which could trigger acute coronary syndrome (ACS). Lastly, the high loss of follow-up rate possibly affected the quality of long-term prognosis and the predictive value of the modified Framingham score.

## Conclusion

Our data suggested that the simple MFS risk stratification tool, which is available in most primary-level hospitals or clinics, showed good performance in estimating the pretest probability of OCAD and identified more than two thirds of relatively stable patients with suspected CAD at high risk for OCAD. Nevertheless, further external validation in larger studies is warranted.

## Additional files

Additional file 1: Figure S1. Study flow. (PDF 54 kb)

Additional file 2: Table S1. Baseline Characteristics and incidence of obstructive coronary artery disease for patients with and without miss data of variables included in the final model. (DOCX 32 kb)

Additional file 3: Table S2. Multivariate logistic regression for the final modified Framingham model of complete and imputation data. (DOCX 19 kb)

Additional file 4: Table S3. Receiver operating characteristic curve for the final modified Framingham model of complete and imputation data. (DOCX 16 kb)

## Abbreviations

CABG: Coronary artery bypass grafting
CAD: Coronary artery disease
CAG: Coronary angiography
CEI/ARB: Angiotensin-converting enzyme inhibitor/angiotensin receptor blocker
CHO: Total cholesterol
CT: Computed tomography
HbA1c: Glycated hemoglobin
HDL-C: High-density lipoprotein cholesterol
Hs-CRP: High-sensitivity C-reactive protein
LDL-C: Low-density lipoprotein-cholesterol
LVEF: Left ventricular ejection fraction
MFS: Framingham scoring
MI: Myocardial infarctions
OCAD: Obstructive coronary artery disease
PCI: percutaneous coronary intervention
TC: Total cholesterol
