# Supplementary material for: A simple prediction model to estimate obstructive coronary artery disease
Source: BMC Cardiovasc Disord. 2018 Jan 16;18:7. doi: 10.1186/s12872-018-0745-0 (PMC5771201; doi:10.1186/s12872-018-0745-0)
Supplement: Supplementary file 1 — Study flow. (PDF 54 kb) [file 12872_2018_745_MOESM1_ESM.pdf]

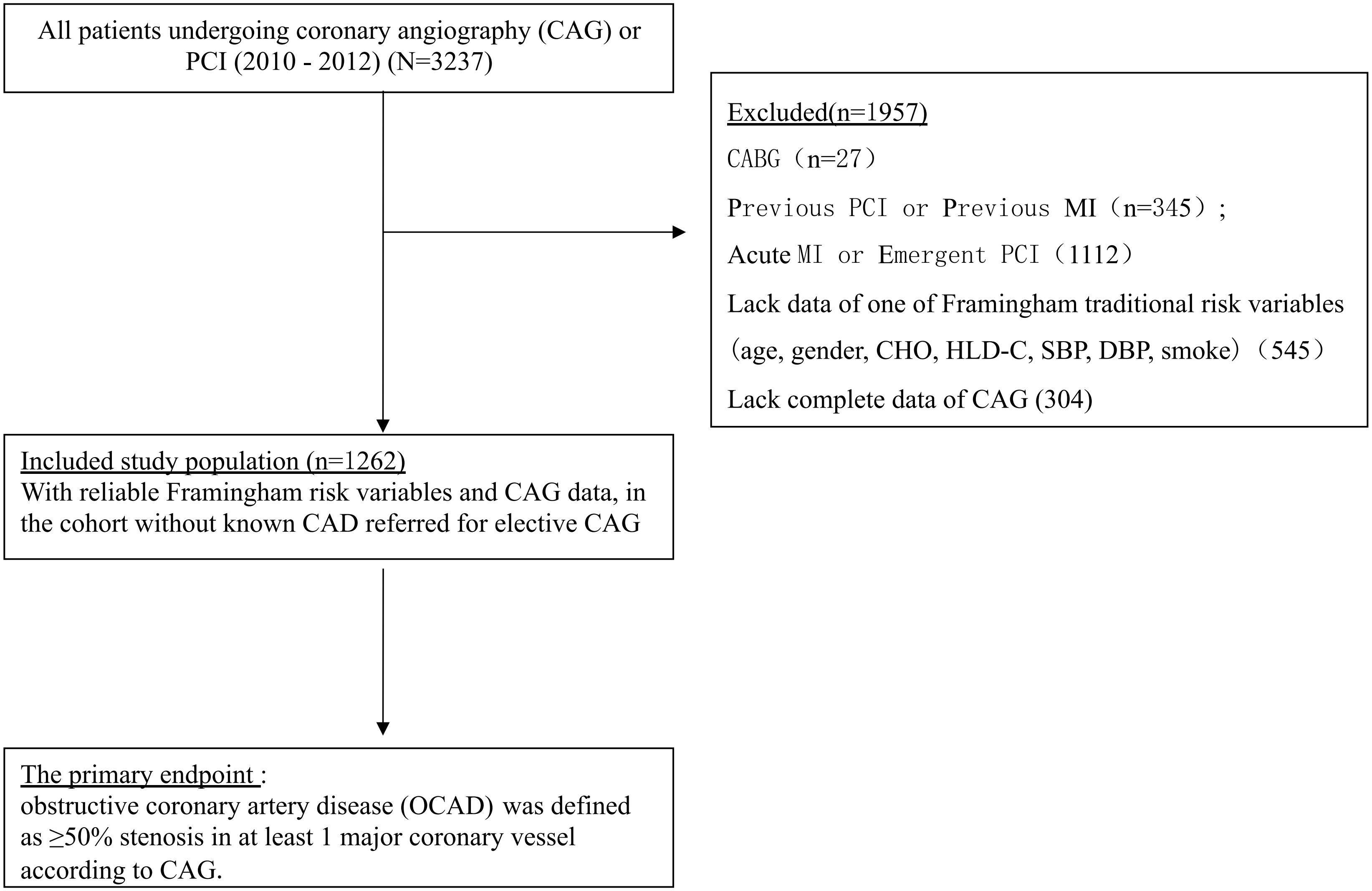
All patients undergoing coronary angiography (CAG) or PCI (2010 - 2012) (N=3237)
Excluded(n=1957)
CABG（n=27）
Previous PCI or Previous MI（n=345）;
Acute MI or Emergent PCI（1112）
Lack data of one of Framingham traditional risk variables （age, gender, CHO, HLD-C, SBP, DBP, smoke）（545）
Lack complete data of CAG (304)
Included study population (n=1262)
With reliable Framingham risk variables and CAG data, in the cohort without known CAD referred for elective CAG
The primary endpoint :
obstructive coronary artery disease (OCAD) was defined as ≥50% stenosis in at least 1 major coronary vessel according to CAG.
