# Supplementary material for: A simple prediction model to estimate obstructive coronary artery disease
Source: BMC Cardiovasc Disord. 2018 Jan 16;18:7. doi: 10.1186/s12872-018-0745-0 (PMC5771201; doi:10.1186/s12872-018-0745-0)
Supplement: Supplementary file 2 — Baseline Characteristics and incidence of obstructive coronary artery disease for patients with and without miss data of variables included in the final model. (DOCX 32 kb) [file 12872_2018_745_MOESM2_ESM.docx]

Supplement Table 1. Baseline Characteristics and incidence of obstructive coronary artery disease for patients with and without miss data of variables included in the final model.

| Variables | Subjects With  miss data of variables included in the final model (n=579） | Subjects Without  miss data of variables included in the final model (n=683） | P value |
| --- | --- | --- | --- |
| Demographic |  |  |  |
| Age | 62.8±10.6 | 64.3±10.0 | 0.01 |
| Gender, men | 412(71.2%) | 462(67.6%) | 0.18 |
| Signs and measurement |  |  |  |
| Heart rate, beats/min | 73.4±11.9 | 72.7±11.7 | 0.32 |
| Systolic BP, mm Hg | 131.3±18.2 | 133.5±18.6 | 0.04 |
| Diastolic BP, mm Hg | 77.7±11.8 | 77.7±11.6 | 0.91 |
| Weight, kg | 65.5±10.6 | 64.1±10.6 | 0.02 |
| Medical history |  |  |  |
| Smoke | 196(33.9%) | 231(33.8%) | 0.99 |
| Hypertension | 336(58.0%) | 431(63.1%) | 0.07 |
| Congestive Heart failure | 45(8.0%) | 53(7.8%) | 0.91 |
| Diabetes mellitus | 133(23.0%) | 177(26.0%) | 0.23 |
| hyperlipidemia | 82(14.2%) | 104(15.2%) | 0.28 |
| Anemia | 173(30.8%) | 255(37.3%) | 0.02 |
| Medications |  |  |  |
| ACEI/ARB | 499(86.2%) | 587(85.9%) | 0.90 |
| Diuretics | 43(7.4%) | 84(12.3%) | <0.01 |
| β-block | 501(86.5%) | 596(87.3%) | 0.70 |
| Statin | 545 (94.1%) | 646(94.6%) | 0.73 |
| Calcium-channel blocker | 112(19.4%) | 146(21.4%) | 0.40 |
| Physical examination |  |  |  |
| LVEF, % | 62.6±11.2 | 62.1±12.0 | 0.53 |
| Laboratory measures |  |  |  |
| Total cholesterol, mg/dl | 169±44.1 | 171.2±41.9 | 0.36 |
| HDL-C，mg/dl | 37.7±9.8 | 37.5±11.1 | 0.80 |
| LDL cholesterol, μmol/l | 2.6±0.9 | 2.6±0.9 | 0.61 |
| Lp (a), μmol/l | 269.3±288.3 | 268.2±300.7 | 0.94 |
| Blood urea nitrogen, mg/dl | 5.1±2.3 | 5.0±2.0 | 0.37 |
| Serum Creatinine, μmol/l | 86.8±42.4 | 86.7±49.9 | 0.97 |
| Hemoglobin, g/ml | 134.6±16.4 | 133.1±15.2 | 0.12 |
| Serum albumin, g/l | 36.5±4.0 | 36.14±4.0 | 0.18 |
| Urine PH | 6.0±0.7 | 6.0±0.7 | 0.70 |
| HbA1c, % | 6.5±1.2 | 6.5±1.2 | 0.53 |
| Hs-CRP, mmol/l | 3.5±4.1 | 6.5±12.9 | <0.001 |
| Uric acid, mmol/l | 382.3±99.0 | 384.3±102.3 | 0.76 |
| B-type Natriuretic Peptide, pg/m | 727.2±2091 | 736.7±2328 | 0.95 |
| Angiograph outcome |  |  |  |
| OCAD | 425(73.4%) | 520(76.1%) | 0.26 |

LVEF = Left Ventricular Ejection Fraction; ACEI/ARB = Angiotensin-Converting Enzyme Inhibitor/Angiotensin Receptor Blocker; HDL-C = High-Density Lipoprotein-Cholesterol; LDL-C = Low-Density Lipoprotein-Cholesterol; HbA1c = Glycated Haemoglobin; Hs-CRP = high-sensitivity C-reactive protein; OCAD: obstructive coronary artery disease.
