# Supplementary material for: A simple prediction model to estimate obstructive coronary artery disease
Source: BMC Cardiovasc Disord. 2018 Jan 16;18:7. doi: 10.1186/s12872-018-0745-0 (PMC5771201; doi:10.1186/s12872-018-0745-0)
Supplement: Supplementary file 3 — Multivariate logistic regression for the final modified Framingham model of complete and imputation data. (DOCX 19 kb) [file 12872_2018_745_MOESM3_ESM.docx]

Supplementary Table 2. Multivariate logistic regression for the final modified Framingham model of complete and imputation data.

| Risk factors | Complete case, n=683^a^ | | | MCMC imputation, n=1262^b^ | | | FCS imputation, n=1262^b^ | | |
| --- | --- | --- | --- | --- | --- | --- | --- | --- | --- |
|  | OR | 95% CI | P value | OR | 95% CI | P value | OR | 95% CI | P value |
| Age (per year) | 1.02 | 1.00-1.04 | 0.05 | 1.02 | 1.01-1.04 | <0.01 | 1.02 | 1.01-1.04 | <0.01 |
| Gender (male vs. female) | 2.99 | 1.97–4.54 | <0.001 | 2.49 | 1.84-3.37 | <0.001 | 2.50 | 1.85-3.38 | <0.001 |
| Hypertension (yes vs. no) | 1.72 | 1.17-2.55 | <0.01 | 2.14 | 1.61-2.83 | <0.001 | 2.13 | 1.61-2.82 | <0.001 |
| Anemia (yes vs. no) | 1.76 | 1.14-2.72 | 0.01 | 1.61 | 1.17-2.20 | <0.01 | 1.61 | 1.17-2.20 | <0.01 |
| LVEF (per %) | 0.98 | 0.96-1.00 | 0.01 | 0.98 | 0.96-0.99 | <0.01 | 0.98 | 0.96-0.99 | <0.01 |
| Hs-CRP (per mmol/l) | 1.03 | 1.00-1.05 | 0.07 | 1.02 | 1.00-1.04 | 0.11 | 1.02 | 1.00-1.04 | 0.10 |
| TC (per mg/dl) | 1.01 | 1.00-1.01 | 0.03 | 1.01 | 1.00-1.01 | <0.001 | 1.01 | 1.00-1.01 | <0.001 |
| HDL-C (per mg/dl) | 0.98 | 0.96-0.99 | <0.01 | 0.97 | 0.96-0.99 | <0.001 | 0.97 | 0.96-0.99 | <0.001 |

MCMC: Markov chain Monte Carlo; FCS：fully conditional specification；OR: odds ratio; CI: confidence interval; LVEF = left ventricular ejection fraction; Hs-CRP = high-sensitivity C-reactive protein; TC: total cholesterol; HDL-C = high-density lipoprotein cholesterol

^a^683 patients without missing data of the variables in the final model

^b^1262 patients with missing data of the variables in the final model
