# Supplementary material for: A simple prediction model to estimate obstructive coronary artery disease
Source: BMC Cardiovasc Disord. 2018 Jan 16;18:7. doi: 10.1186/s12872-018-0745-0 (PMC5771201; doi:10.1186/s12872-018-0745-0)
Supplement: Supplementary file 4 — Receiver operating characteristic curve for the final modified Framingham model of complete and imputation data. (DOCX 16 kb) [file 12872_2018_745_MOESM4_ESM.docx]

Supplementary Table 3. Receiver operating characteristic curve for the final modified Framingham model of complete and imputation data

| Receiver operating characteristic curve | Complete case, n=683^a^ | | MCMC imputation, n=1262^b^ | | FCS imputation, n=1262^b^ | |
| --- | --- | --- | --- | --- | --- | --- |
|  | Value | 95% CI | Mean | Range | Mean | Range |
| C-statistic | 0.719 | 0.672-0.766 | 0.712±0.003 | 0.706-0.721 | 0.711±0.003 | 0.704-0.720 |

MCMC: Markov chain Monte Carlo；FCS：fully conditional specification；CI: confidence interval

^a^683 patients without missing data of the variables in the final model

^b^1262 patients with missing data of the variables in the final model
